# Evidence for the Existence of Autotrophic Nitrate-Reducing Fe(II)-Oxidizing Bacteria in Marine Coastal Sediment

**DOI:** 10.1128/AEM.01570-16

**Published:** 2016-09-30

**Authors:** Katja Laufer, Hans Røy, Bo Barker Jørgensen, Andreas Kappler

**Affiliations:** a Geomicrobiology, Center for Applied Geosciences, University of Tübingen, Tübingen, Germany; b Center for Geomicrobiology, Department of Bioscience, Aarhus University, Aarhus, Denmark; Georgia Institute of Technology

## Abstract

Nitrate-reducing Fe(II)-oxidizing microorganisms were described for the first time ca. 20 years ago. Most pure cultures of nitrate-reducing Fe(II) oxidizers can oxidize Fe(II) only under mixotrophic conditions, i.e., when an organic cosubstrate is provided. A small number of nitrate-reducing Fe(II)-oxidizing cultures have been proposed to grow autotrophically, but unambiguous evidence for autotrophy has not always been provided. Thus, it is still unclear whether or to what extent Fe(II) oxidation coupled to nitrate reduction is an enzymatically catalyzed and energy-yielding autotrophic process or whether Fe(II) is abiotically oxidized by nitrite from heterotrophic nitrate reduction. The aim of the present study was to find evidence for the existence of autotrophic nitrate-reducing Fe(II) oxidizers in coastal marine sediments. Microcosm incubations showed that with increasing incubation times, the stoichiometric ratio of reduced nitrate/oxidized Fe(II) [NO_3_^−^_reduced_/Fe(II)_oxidized_] decreased, indicating a decreasing contribution of heterotrophic denitrification and/or an increasing contribution of autotrophic nitrate-reducing Fe(II) oxidation over time. After incubations of sediment slurries for >10 weeks, nitrate-reducing activity ceased, although nitrate was still present. This suggests that heterotrophic nitrate reduction had ceased due to the depletion of readily available organic carbon. However, after the addition of Fe(II) to these batch incubation mixtures, the nitrate-reducing activity resumed, and Fe(II) was oxidized, indicating the activity of autotrophic nitrate-reducing Fe(II) oxidizers. The concurrent reduction of ^14^C-labeled bicarbonate concentrations unambiguously proved that autotrophic C fixation occurred during Fe(II) oxidation and nitrate reduction. Our results clearly demonstrated that autotrophic nitrate-reducing Fe(II)-oxidizing bacteria were present in the investigated coastal marine sediments.

**IMPORTANCE** Twenty years after the discovery of nitrate-reducing Fe(II) oxidizers, it is still controversially discussed whether autotrophic nitrate-reducing Fe(II)-oxidizing microorganisms exist and to what extent Fe(II) oxidation in this reduction/oxidation process is enzymatically catalyzed or which role abiotic side reactions of Fe(II) with reactive N species play. Most pure cultures of nitrate-reducing Fe(II) oxidizers are mixotrophic; i.e., they need an organic cosubstrate to maintain their activity over several cultural transfers. For the few existing autotrophic isolates and enrichment cultures, either the mechanism of nitrate-reducing Fe(II) oxidation is not known or evidence for their autotrophic lifestyle is controversial. In the present study, we provide evidence for the existence of autotrophic nitrate-reducing Fe(II) oxidizers in coastal marine sediments. The evidence is based on stoichiometries of nitrate reduction and Fe(II) oxidation determined in microcosm incubations and the incorporation of carbon from CO_2_ under conditions that favor the activity of nitrate-reducing Fe(II) oxidizers.

## INTRODUCTION

Iron is an abundant redox-active element that makes up about 5% of the earth's crust (1). In the environment, the two most important redox states of iron are Fe(II) (ferrous iron) and Fe(III) (ferric iron). In the biogeochemical Fe cycle, different biotic and abiotic reactions are involved in the oxidation of Fe(II) to Fe(III) or the reduction of Fe(III) to Fe(II). At circumneutral pH, the most important abiotic reactions include the oxidation of Fe(II) by O_2_, reactive N species, or Mn(IV) minerals as well as the reduction of Fe(III) by reduced sulfur species, humic substances, or light-induced reactions (2). Microbial Fe-converting metabolic processes include the oxidation of Fe(II) by microaerophilic (3), anoxygenic phototrophic (4), and nitrate-reducing (5) Fe(II) oxidizers as well as the reduction of Fe by Fe(III)-reducing microorganisms that either live heterotrophically and use organic carbon as an electron donor or live autotrophically and use hydrogen as an electron donor (6, 7).

Iron is an important element, not only because it can be used as an electron acceptor or electron donor by many different microbes that gain energy from that reaction but also because the Fe cycle is connected to many other elementary cycles, such as the C and N cycles (3–5, 8–10). Furthermore, the speciation of Fe and the properties of the Fe minerals that are formed, transformed, or dissolved by microbial activity or by abiotic reactions can influence the fate of nutrients, pollutants, and trace metals (11–16). Unfortunately, there are knowledge gaps that hamper our understanding of the biogeochemical Fe cycle, mainly regarding the mechanisms of Fe(II) oxidation in general and particularly nitrate-reducing Fe(II) oxidation. The energetic yield of the reaction would allow autotrophic growth (equation 1) (17), but now, 20 years after the first discovery of organisms that concurrently reduce nitrate and oxidize Fe(II) (5), it is still unclear whether, or to which extent, this process is enzymatically catalyzed and if bacteria can gain energy from this thermodynamically favorable reaction:

(1) $$\begin{array}{c} {10\text{Fe}}^{2+}+2{\text{NO}}_{3}^{-}+24{\text{H}}_{2}\text{O}\to 10\text{Fe}{\left(\text{OH}\right)}_{3}+{\text{N}}_{2}+{18\text{H}}^{+}\\ \Delta G{}^{\circ}\prime =\;-96.23\;\text{kJ}/\text{mol Fe} \end{array}$$

Most known cultures of nitrate-reducing Fe(II) oxidizers cannot be cultivated autotrophically over a long period or over several transfers with nitrate and Fe(II) alone but need an organic cosubstrate (18–22). Particularly for these mixotrophic cultures, it is unknown whether Fe(II) oxidation is enzymatically catalyzed and coupled to cell metabolism or whether Fe(II) oxidation is merely an abiotic side reaction caused by reactive N intermediates of heterotrophic nitrate reduction (23–26). An indication for an unintended abiotic side reaction is that most known mixotrophic nitrate-reducing Fe(II)-oxidizing cells become encrusted in Fe(III) minerals during Fe(II) oxidation (26), a process that is potentially harmful for cells (27). In contrast, microaerophilic or phototrophic autotrophic Fe(II) oxidizers that enzymatically oxidize Fe(II) have developed strategies to avoid the encrustation of cells (27–30), and it is remarkable that no such strategies have been demonstrated to be present in nitrate-reducing Fe(II) oxidizers. Furthermore, it appears that the ability for Fe(II) oxidation is common among all nitrate-reducing bacteria, when an organic substrate is provided (24, 26), even for Escherichia coli (31). Nevertheless, there are a few strains with the proposed ability to live autotrophically with only nitrate and Fe(II): Pseudogulbenkiania sp. strain 2002 (32), Ferroglobus placidus (33), Paracoccus ferrooxidans BDN-1 (34), Thiobacillus denitrificans (5), Geobacter metallireducens (35), Desulfitobacterium frappieri strain G2 (36), Citrobacter freundii strain PXL1 (37), and Mycobacterium sp. strain W5 (38). In addition to these pure cultures, there is the nitrate-reducing Fe(II)-oxidizing enrichment culture KS (5, 39), which is a coculture of several microbial strains (5, 39) that grows autotrophically and can be transferred continuously with only Fe(II) and nitrate. However, the mechanism of Fe(II) oxidation, and also which strain in the coculture is oxidizing Fe(II), is still unknown (39). For most of the above-mentioned “autotrophic” cultures, except for the KS enrichment culture, unambiguous evidence for their ability to oxidize Fe(II) autotrophically with nitrate [such as data for repeatedly transferred cultures and data for CO_2_ fixation coupled to Fe(II) oxidation and nitrate reduction, etc.] is still missing, and no enzymes involved in Fe(II) oxidation by nitrate-reducing Fe(II) oxidizers have been identified.

Additionally, no evidence for the actual occurrence of autotrophic nitrate-reducing Fe(II) oxidation in the environment has been presented so far. Nitrate-reducing Fe(II)-oxidizing bacteria were found in many different environments, including soils, freshwater sediments, marine sediments, hypersaline sediments, and hydrothermal vents (5, 33, 40–44). Furthermore, the potential activity of nitrate-reducing Fe(II) oxidizers has been investigated in enrichment cultures from wetland sediment or natural sediment from river floodplains, lake sediment, marine sediment, or soil (40, 42, 45–49). However, the nitrate-dependent Fe(II) oxidation observed in most of these setups was probably mixotrophic.

The aim of the present study, therefore, was to find evidence for the presence of autotrophic nitrate-reducing Fe(II) oxidizers in the environment. This was achieved by quantification of Fe(II) oxidation and nitrate reduction rates in microcosm incubations with coastal marine sediments from Norsminde Fjord and Kalø Vig, Denmark, before and after readily available organic carbon had been depleted. Additionally, we quantified the incorporation of ^14^C-labeled bicarbonate in microcosms with sediment from Norsminde Fjord under conditions that favor the activity of autotrophic nitrate-reducing Fe(II)-oxidizing bacteria. For the ^14^C incorporation study, we chose the sediment from the Norsminde Fjord field site, because we previously characterized this field site and showed active microbial Fe cycling in this sediment (49).

## MATERIALS AND METHODS

### Study site.

In June 2015, sediment was sampled at Norsminde Fjord (Denmark), a shallow brackish estuary with freshwater inflow mainly through the Odder River in the western part and a narrow opening to the Baltic Sea in the eastern part. Samples from Norsminde Fjord were taken near the narrow entrance to the Baltic Sea in the southeastern part of the fjord (56°01.171′N, 010°15.390′E). The material collected from Norsminde Fjord was organic-rich mud. In February 2015, sediment was sampled at a beach at Kalø Vig, Denmark (56°16.811′N, 010°28.056′E), at a 0.5- to 1-m water depth. At Kalø Vig, the collected material was an organic-poor silty-sand sediment.

### Sediment sampling.

Sediment for microcosm incubations was sampled in core liners with a 2.5-cm inner diameter (50-ml syringes cut off at the front ends). The uppermost 3 cm of several sediment cores was pooled and homogenized. Any visible stones, animals, or other organic-rich particles were removed from the sediment by using tweezers.

### Geochemical measurements.

Sediment, pore water, and surface water characteristics were determined by a combination of field measurements with a field multimeter (WTW Multi 3430 for measurement of oxygen saturation, pH, temperature, and salinity of the water column) and microelectrode measurements (glass electrodes with a 100-μm tip diameter [Unisense, Denmark] for measurement of oxygen and sulfide concentrations as well as pH and redox potential over sediment depth in the first few millimeters of the sediment). Microelectrode measurements were performed on freshly sampled sediment cores directly at the field site. We determined sediment water and total organic carbon (TOC) contents and pore water concentrations of dissolved organic carbon (DOC), nitrate, nitrite, Fe(II), and Fe(III) as previously described by Laufer et al. (44).

### MPN enumeration of nitrate-reducing Fe(II)-oxidizing and Fe(III)-reducing bacteria.

Enumeration of Fe-metabolizing microorganisms was done with the original sediments used for the preparation of the microcosms (homogenized sediment from a 0- to 3-cm sediment depth). Numbers of viable cells of mixotrophic nitrate-reducing Fe(II) oxidizers and Fe(III)-reducing bacteria were determined according to the most probable number (MPN) method with artificial seawater (ASW) medium as described previously by Laufer et al. (44). It was not possible to use the MPN method to determine viable cell numbers of autotrophic nitrate-reducing Fe(II)-oxidizing bacteria due to the high organic carbon content of the sediments used for inoculation of the MPNs, which favored mixotrophic nitrate-reducing Fe(II) oxidizers and prevented us from separately quantifying autotrophs in these sediments.

### Microcosm incubations with Norsminde Fjord sediment. (i) Preincubation and depletion of bioavailable organic carbon.

**FIG 1:**
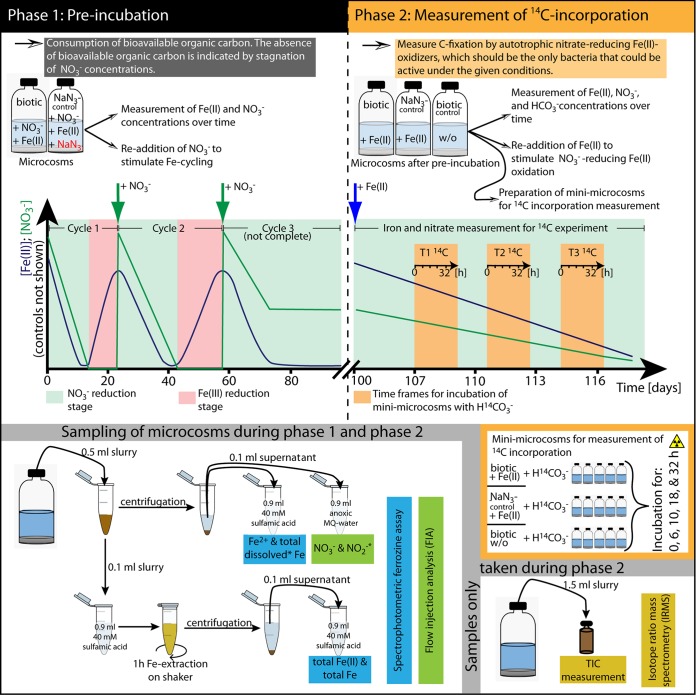
Schematic representation of the experimental setup. (Top) The microcosm experiment was divided into two phases. The purpose of the first phase was to deplete the microcosms from bioavailable organic carbon to be able to quantify the incorporation of ^14^CO_2_ in the second phase by autotrophic nitrate-reducing Fe(II) oxidizers. (Bottom) Sampling procedure. Asterisks indicate parameters that were measured only during the first phase or that were measured by a different method in the second phase. MQ, Milli-Q water.

The experimental setup for the microcosm incubations is shown in Fig. 1. In the first phase, the sediment was preincubated in the presence NO_3_^−^ and Fe(III)/Fe(II) in order to starve the microcosms of readily available organic carbon. Preincubation (phase 1) was composed of three iron redox cycles. In the first redox cycle, in the Fe(II) oxidation stage, NO_3_^−^ was reduced by heterotrophs and autotrophs, while Fe(II) was oxidized until NO_3_^−^ was depleted. The following Fe(III) reduction stage was initiated by the depletion of NO_3_^−^. Now, the heterotrophic activity shifted to Fe(III) reduction until all reactive iron was converted to Fe(II). The second and third redox cycles were then initiated by the readdition of NO_3_^−^ (Fig. 1). Cyclic preincubation was continued until NO_3_^−^ was no longer depleted, indicating that all readily available organic carbon was depleted.

### (ii) ^14^C incorporation experiments.

The main incubation (phase 2) was started after 103 days by the readdition of Fe(II) to the microcosms, after which NO_3_^−^ reduction resumed. As NO_3_^−^ was the only available electron acceptor and Fe(II) was the only available electron donor in this carbon-starved system, the process of nitrate-reducing Fe(II) oxidation was expected to happen autotrophically. Autotrophic CO_2_ fixation during the main incubation was quantified via ^14^C incorporation in separate subsamples (mini-microcosms) (Fig. 1).

### (iii) Preparation, incubation, and sampling of microcosms.

All preparation steps for the microcosms were performed in an anoxic glove box (100% N_2_ atmosphere). Microcosm incubations were set up with 100-ml serum vials that were wrapped twice with aluminum foil to keep them dark. Five grams of the homogenized sediment and 50 ml seawater medium were used for each microcosm. Seawater that was sampled at the field site was made anoxic by flushing with N_2_ for 1 h per liter water, filtering the seawater through a 0.22-μm filter (polyethersulfone [PES]) (Steritop; EMD Millipore), replacing the headspace with N_2_-CO_2_ (90:10), and adding 20 mM MOPS [3-(*N*-morpholino)propanesulfonic acid] as a buffer. One milliliter of vitamin solution (50), 1 ml trace-element solution (51), and 1 ml selenite-tungstate solution (52) were added for each liter of seawater medium. An anoxic and sterile Na_2_MoO_4_ solution was added to a final concentration of 20 mM to inhibit the activity of sulfate-reducing bacteria (53, 54). The pH of the medium was adjusted to 7.1 and regularly checked during incubation. The following additives (all sterile and anoxic) were added to the microcosms in the beginning of the first phase: FeCl_2_ (2 mM) and NaNO_3_ (4 mM). In total, 32 replicates of this treatment were prepared. Nine inhibited controls were prepared with the same addition of FeCl_2_ and NaNO_3_, supplemented with 160 mM NaN_3_. The efficiency of NaN_3_ inhibition was evaluated with several enrichment cultures of nitrate-reducing Fe(II) oxidizers and Fe(III) reducers at different concentrations of NaN_3_ (1 to 160 mM), and no Fe(II) oxidation or Fe(III) reduction was detected in these tests (see Table S1 in the supplemental material).

All microcosms were incubated at 20°C in the dark in sets of 3 replicates. In the preincubation phase of the experiment, we monitored Fe(II) and Fe(III) concentrations in the solid fraction of the slurry (extraction with 40 mM sulfamic acid, which extracts poorly crystalline Fe phases [23]) and Fe(II)/Fe(III) plus NO_3_^−^ and NO_2_^−^ concentrations in the dissolved phase. After 23 and 57 days, 4 mM NO_3_^−^ was readded to all microcosms to initiate the second and third Fe redox cycles, respectively (Fig. 1, top left).

After 103 days of incubation, the second phase of the experiment was started. Before that, the Fe(II) concentration remained stable at ca. 0.6 mM after day 71, and no further NO_3_^−^ reduction was observed after day 71. To start the second phase, 2 mM FeCl_2_ was added to the following preincubated microcosms from the first phase: (i) six microcosms that were preincubated with Fe(II) and NO_3_^−^ and (ii) six control microcosms that were preincubated with Fe(II), NO_3_^−^, and NaN_3_ (inhibited control). As a control for background C fixation without an involvement of Fe(II) oxidation, six preincubated microcosms from phase 1 were incubated without any amendments (background C fixation control). In the second phase, the Fe(II) and Fe(III) concentrations in the slurry and concentrations of NO_3_^−^ in the dissolved phase were monitored. Additional samples were taken for the quantification of total inorganic carbon (TIC) concentrations. At three time points during the second phase of the experiment, mini-microcosms for the quantification of ^14^C incorporation were prepared from the original microcosms (see below).

### (iv) Preparation, incubation, and sampling of mini-microcosms.

At three time points (after 105, 109, and 113 days of incubation) during the second phase of the experiment, mini-microcosms were prepared from subsamples of the original microcosms. For the mini-microcosms, 10-ml glass vials were sterilized and closed with a butyl stopper and an aluminum crimp cap, and the headspace was flushed with N_2_-CO_2_ gas. The vials were wrapped twice with aluminum foil to exclude light. Five milliliters of the slurry was then sampled anoxically from the respective microcosm with a syringe and a thick hypodermic needle (1.2 by 40 mm) and injected into anoxic, sterile glass vials. Afterwards, the mini-microcosms were incubated overnight before the radioactive ^14^C tracer was added. At each of the three time points, seven mini-microcosms were prepared from (i) microcosms preincubated with Fe(II) and NO_3_^−^, to which no additives were added at the beginning of the second phase (to measure background C fixation); (ii) microcosms that were preincubated with Fe(II) and NO_3_^−^, to which Fe(II) was added at the beginning of the second phase; and (iii) control microcosms that were preincubated with Fe(II), NO_3_^−^, and NaN_3_, to which Fe(II) was added at the beginning of the second phase (inhibited control). To each mini-microcosm, 25 kBq of a carrier-free H^14^CO_3_^−^ tracer was added. The mini-microcosms were incubated with the ^14^C tracer for different time periods ranging from 0 to 32 h (0, 6, 10, 18, and 32 h). The incubations were stopped by adding 0.25 ml of a 0.5 M Na_2_CO_3_ solution (to bring the pH above 9 and avoid the loss of nonfixed ^14^CO_2_ to the atmosphere when the vials are opened later) and freezing the mixtures at −20°C. For the microcosms that were incubated for 0 h, incubation was stopped within 1 to 2 min after the addition of the tracer. One mini-microcosm was sampled for each treatment at each time interval except after 18 h, when triplicate mini-microcosms were sampled.

### Microcosm experiment with Kalø Vig sediment.

Sampling and preparation of microcosms with Kalø Vig sediment were done similarly to those of the microcosms with sediment from Norsminde Fjord. Microcosms were prepared in 100-ml serum vials, to which 5 g of homogenized sediment and 50 ml seawater medium (anoxic and sterile filtered) that was buffered with 20 mM MOPS buffer were added. The same vitamins and trace-element solutions as those used in the Norsminde Fjord medium were added. FeCl_2_ and NO_3_^−^ were both added at a starting concentration of 2 mM. Control incubation mixtures were treated with 160 mM NaN_3_ (inhibited control). Similarly to the Norsminde Fjord microcosms, the concentrations of Fe(II) and total Fe were analyzed over time in the solid phase (40 mM sulfamic acid extraction) and the dissolved phase. Also, the concentrations of NO_3_^−^ and NO_2_^−^ were monitored over time by using the same analytical techniques as those used on the Norsminde Fjord microcosms. After 32 days, 2 mM FeCl_2_ was readded to all microcosms.

### Analytical techniques and calculations. (i) Quantification of Fe(II), Fe(III), NO_3_^−^, NO_2_^−^, and TIC concentrations in microcosms.

Quantification of concentrations of Fe(II) and total Fe in the dissolved phase and of sulfamic acid-extractable Fe was performed by using a ferrozine assay (55). Sampling was performed as shown in Fig. 1. A flow injection analysis (FIA) instrument equipped with a dialysis membrane for removal of Fe to prevent side reactions during measurement (Seal Analytical, Germany) was applied for quantification of NO_3_^−^ and NO_2_^−^ concentrations. Fe(II) and total Fe concentrations in the dissolved phase were quantified only in the first phase of the experiment. In the second phase, only Fe(II) and total Fe concentrations in the sulfamic acid-extractable Fe fraction and NO_3_^−^ concentrations in the dissolved phase were quantified. Concentrations of NO_2_^−^ in the second phase of the experiment were evaluated with indicator sticks (MQuant nitrite test, with a detection limit of 2 mg NO_2_^−^ liter^−1^, equal to 40 μM; Merck). In the second phase, additional samples for quantification of TIC concentrations were taken. Therefore, 1.5 ml of the slurry was sampled with a syringe and needle and transferred into a small-headspace vial that was completely filled and closed immediately. Samples were stored at 4°C until measurement. For quantification of TIC concentrations, 0.1 ml of the sample was transferred to a sealed Exetainer (Labco Limited, UK) and acidified with 0.05 ml of phosphoric acid (85%). After 24 h of equilibration, the evolved CO_2_ was measured in the headspace of the Exetainer by isotope ratio mass spectrometry (IRMS) (Delta V Plus; Thermo).

### (ii) Calculation of rates of Fe(II) oxidation, Fe(III) reduction, and NO_3_^−^ reduction and stoichiometries of NO_3_^−^_reduced_ versus Fe(II)_oxidized_.

Rates of Fe(II) oxidation, Fe(III) reduction, and NO_3_^−^ reduction were calculated by linear regression of the measured concentrations of the respective compounds over time at the highest rate of increasing or decreasing concentrations. At least 3 time points were used for each rate calculation. The stoichiometries of reduced NO_3_^−^ (NO_3_^−^_reduced_) versus oxidized Fe(II) [Fe(II)_oxidized_] during different time intervals were calculated by dividing the amount of nitrate that was reduced during that time interval (calculated from the total decrease in nitrate concentrations, including nonlinear segments) by the amount of Fe(II) that was oxidized during that same time interval.

### Incubations with H^14^CO_3_^−^ and measurement of ^14^C incorporation into biomass. (i) Determination of ^14^C incorporation into biomass via wet fumigation.

Fixation of H^14^CO_3_^−^ into biomass was quantified via wet fumigation according to a method described previously by Ji et al. (56), with modifications to make it suitable for sediment samples and an inorganic ^14^C tracer. First, the slurry from the mini-microcosm was transferred into a Falcon tube (15 ml) and centrifuged (5 min at 5,000 × *g*). For quantification of the amount of unreacted ^14^C tracer (counts per minute of dissolved inorganic carbon [cpm_DIC_]), 100 μl of the supernatant was added to a scintillation vial containing 900 μl CarboSorbE (PerkinElmer) and 19 ml Permafluor (PerkinElmer) scintillation cocktail. Radioactivity (in counts per minute) was determined with a liquid scintillation counter (Tri-Carb 2900TR; Packard Instruments). The sediment pellet was transferred into one side vial of a custom-made H-shaped glass vessel, of which both openings were closed with butyl rubber stoppers and a screw cap (see Fig. S1 in the supplemental material). A total of 0.5 ml of H_2_SO_3_ was then added to the sediment to decrease the pH to ca. 2, and the headspace of the vessel was flushed for 12 h with N_2_ while stirring the sediment-acid mixture with a magnetic stirrer to remove residual carbonate. The emerging CO_2_ was trapped in CarboSorbE to test if the tracer was lost to crystalline carbonate during incubation. For measurement of the amount of ^14^CO_2_ released after acidification, 100 μl of CarboSorbE was transferred into a scintillation vial containing 900 μl CarboSorbE and 19 ml Permafluor scintillation cocktail, and radioactivity was measured by liquid scintillation counting. To oxidize all organic carbon in the sediment-acid mixture left after acidification, 300 mg of K_2_Cr_2_O_7_ and 5 ml of K_2_Cr_2_O_7_ in an H_2_SO_4_ solution (12 g/100 ml) were added to the sediment and mixed well with the sediment by stirring with a magnetic stirrer. Afterwards, the tubes were autoclaved for 2 h at 121°C and then cooled to room temperature. This treatment with a strong oxidant and heat converts all organic carbon into CO_2_. To trap the organic-derived CO_2_ present in the headspace of the H tube, 1 ml of CarboSorbE was added to the second side tube of the vessel, and the mixtures in both vessels of the H-shaped glass vessel were stirred for 24 h. Finally, CarboSorbE was transferred into a scintillation vial containing 19 ml of Permafluor scintillation cocktail for quantification of the fraction of ^14^C incorporated into organic matter during incubation (cpm_fix_). In addition, the remaining waste from the wet fumigation (mixture of sediment pellet-acid-K_2_Cr_2_O_7_) was prepared for scintillation counting by adding 100 μl of the waste to a scintillation vial containing 900 μl CarboSorbE and 19 ml Permafluor, and radioactivity was determined by liquid scintillation counting.

### (ii) Calculation of ^14^C incorporation rates.

The rates of CO_2_ incorporation per milliliter of slurry per hour at each time point were calculated according to the following equation:

(2) $$\frac{{\text{cpm}}_{\text{fix}}\times 1.05\times \text{DIC}}{({\text{cpm}}_{\text{fix}}+{\text{cpm}}_{DIC})\times t}=\text{C}\;\text{fixed}\;[\text{mmol}\;{\text{ml}}^{-1}\;{\text{h}}^{-1}]$$

where cpm_fix_ is the counts from scintillation counting for ^14^C that was fixed into biomass (corrected for the counts per minute that were already found in the time zero control); cpm_DIC_ is the counts from the supernatants of the respective mini-microcosms by liquid scintillation counting (back-calculated to the total amount of radioactivity in the full volume of the mini-microcosm); 1.05 is a conversion factor, as bacteria preferentially take up the lighter isotope (e.g., see reference 57); DIC is the concentration of inorganic carbon in the microcosm (millimoles per milliliter); and *t* is the time that the mini-microcosms was incubated with the ^14^C tracer.

## RESULTS

### Characterization of the sediment and pore water.

Visual observation of the sediment from Norsminde Fjord showed that it was oxidized in the upper ca. 0.5 cm, indicated by a light brown color. The deeper part of the sediment was black, indicating reducing conditions and the presence of FeS. Oxygen penetrated only 2 mm into the sediment. The DOC concentration in the water column was 4.9 mg liter^−1^, and the TOC content in the sediment was 2.9 wt% [i.e., 2.9% (wt/wt)]. The sediment from Kalø Vig had a light brown color in the uppermost centimeter and was slightly grayish below that. Oxygen penetrated the sediment to about 5 mm. The DOC concentration in the water column was 4.4 mg liter^−1^, and the TOC content in the sediment was 0.45 wt%. Detailed results of the geochemical measurements are shown in Table 1.

**TABLE 1:** Geochemical parameters and MPN of Fe(III) reducers and nitrate-reducing Fe(II) oxidizers in sediment from Norsminde Fjord and Kalø Vig^*a*^

Parameter	Value for sediment	
	Norsminde Fjord	Kalø Vig
% O_2_ saturation of water column	100	100
O_2_ penetration depth in sediment (mm)	2	5
Salinity	22.1	23.3
pH of water column	8.2	7.8
pH in anoxic part of sediment (sediment depth [mm])	7.4 (2)	7.2 (7)
Redox potential in water column (mV)	+380	+435
Redox potential in sediment (mV) (sediment depth [mm])	−98 (5)	+263 (15)
Mean Fe(II) concn in pore water (μmol liter^−1^) ± SD	92 ± 60	23 ± 53
Mean Fe(II) concn in sediment (1 M HCl extraction) (μmol g^−1^ [dry wt]) ± SD	35 ± 21	0.4 ± 0.1
NO_3_^−^ concn in water column (μmol liter^−1^)	64	ND
NO_2_^−^ concn in water column (μmol liter^−1^)	BDL	ND
Mean NO_3_^−^ concn in pore water (μmol liter^−1^) ± SD	BDL	11.66 ± 2.9
NO_2_^−^ concn in pore water (μmol liter^−1^)	BDL	BDL
Mean TOC concn in sediment (%) ± SD	2.9 (per wt)	0.45 ± 0.32
Mean DOC concn in water column (mg liter^−1^) ± SD	4.9	4.4 ± 0.1
Mean MPN NO_3_^−^-reducing Fe(II) oxidizers^*b*^ (cells g^−1^ [dry wt]) (range)	6.9 × 10^3^ (3.2 × 10^3^–8.3 × 10^3^)	5.7 × 10^3^ (2.4 × 10^3^–7.9 × 10^3^)
Mean MPN Fe(III) reducers (cells g^−1^ [dry wt]) (range)	4.7 × 10^3^ (9.8 × 10^2^–6.2 × 10^3^)	2.9 × 10^2^ (1.2 × 10^2^–6.7 × 10^2^)

a BDL, below the detection limit; ND, not determined.

b MPN were determined only for mixotrophic nitrate-reducing Fe(II) oxidizers, as due to the high TOC concentration in the sediment, it was not possible to determine MPN for autotrophic nitrate-reducing Fe(II) oxidizers.

### MPN enumeration of nitrate-reducing Fe(II) oxidizers and Fe(III) reducers.

We found significant numbers of mixotrophic nitrate-reducing Fe(II) oxidizers and Fe(III) reducers in the original sediment from Norsminde Fjord of 6.9 × 10^3^ cells g^−1^ (dry weight) (range, 3.2 × 10^3^ to 8.3 × 10^3^ cells g^−1^ [dry weight]) and 4.7 × 10^3^ cells g^−1^ (dry weight) (range, 9.9 × 10^2^ to 6.2 × 10^3^ cells g^−1^ [dry weight]), respectively (Table 1). In the sediment from Kalø Vig, numbers of viable cells of nitrate-reducing Fe(II) oxidizers and Fe(III) reducers were 5.7 × 10^3^ cells g^−1^ (dry weight) (range, 2.4 × 10^3^ to 7.9 × 10^3^ cells g^−1^ [dry weight]) and 2.9 × 10^2^ cells g^−1^ (dry weight) (range, 1.2 × 10^3^ to 6.8 × 10^2^ cells g^−1^ [dry weight]), respectively (Table 1).

### Fe(II) oxidation and Fe(III) reduction in microcosms over time.

**FIG 2:**
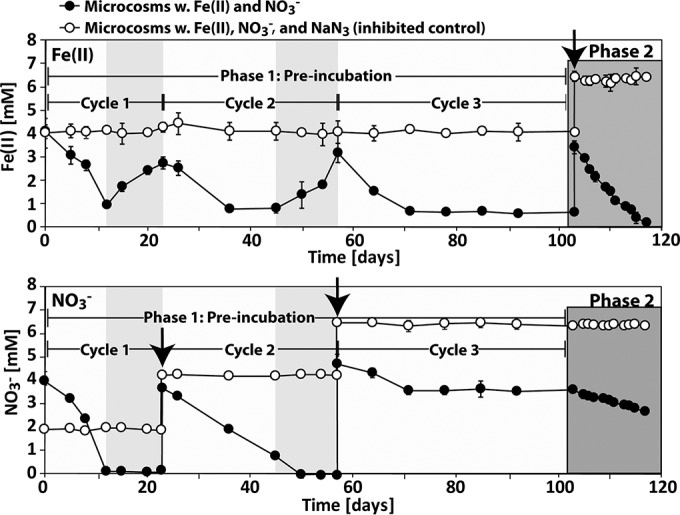
Temporal development of Fe(II) (top) and NO_3_^−^ (bottom) concentrations in the first and second phases of the microcosm experiment with sediment from Norsminde Fjord. Shown are the data for total Fe(II) (extraction with 40 mM sulfamic acid) and NO_3_^−^ in the dissolved phase. In the top panel, the black arrow indicates the time point at which Fe(II) was readded to the microcosms. In the bottom panel, the black arrows indicate the time points at which NO_3_^−^ was readded to the microcosms. Phase 1 is the first phase of the experiment, in which nitrate was added, organic carbon was depleted, and Fe was cycled. The light gray boxes in phase 1 indicate when Fe(III) reduction was occurring. Error bars show standard deviations of data from three replicates.

**FIG 3:**
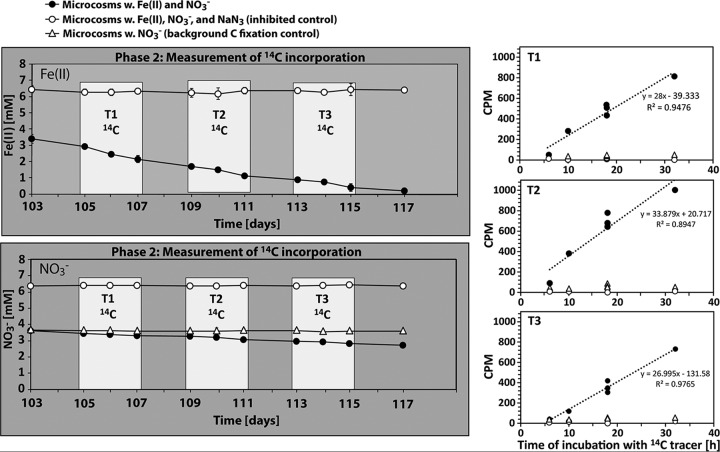
(Left) Temporal development of Fe(II) (top) and NO_3_^−^ (bottom) concentrations in the second phase of the microcosm experiment with sediment from Norsminde Fjord. Shown are the data for total Fe(II) (extraction with 40 mM sulfamic acid) and NO_3_^−^ in the dissolved phase. The incorporation of an inorganic ^14^C tracer (^14^C-labeled bicarbonate) into biomass was quantified in mini-microcosms at three time intervals (T1, T2, and T3) (indicated by the white boxes). Error bars show standard deviations of data from three replicates (in some cases, they are smaller than the symbol). (Right) Results of liquid scintillation counting of ^14^C incorporated into biomass from incubations in mini-microcosms during the three different time intervals.

The microcosm experiment with sediment from Norsminde Fjord was divided into two phases (Fig. 1). The first phase consisted of three oxidation-reduction cycles, the third of which was not complete and consisted of Fe(II) oxidation only. This preincubation was intended to deplete the sediment of organic carbon readily available for heterotrophic processes. The second phase of the experiment consisted of only Fe(II) oxidation and was set up to reveal potential autotrophic nitrate-reducing Fe(II) oxidation. In Fig. 2 and 3, total Fe(II) concentrations are shown for both phases of the microcosm experiment. Table 2 provides an overview of the concentrations of Fe(II) and Fe(III) as well as the rates of Fe(II) oxidation and Fe(III) reduction. The extents of Fe(II) oxidation and Fe(III) reduction vary in different cycles. The rates of Fe(III) reduction were comparable (0.16 and 0.18 mM day^−1^) in redox cycles 1 and 2. Rates of Fe(II) oxidation decreased from cycle 1 (0.26 mM day^−1^) to cycle 2 (0.15 mM day^−1^) and increased slightly again in cycle 3 (0.19 mM day^−1^) (Table 2).

**TABLE 2:** Decreases of Fe(II) and NO_3_^−^ concentrations and rates and stoichiometries of Fe(II) oxidation, Fe(III) reduction, and NO_3_^−^ reduction in the two phases of the microcosm experiment with sediment from Norsminde Fjord

Parameter	Value for expt			
	Phase 1			Phase 2
	Cycle 1	Cycle 2	Cycle 3	
Decrease of Fe(II) concn (mM)	3.15	1.98	2.59	3.20
Decrease of Fe(III) concn (mM)	1.79	2.41	—^*a*^	—^*a*^
Decrease of NO_3_^−^ concn (mM)	3.87	3.69 (−1.74^*b*^)	1.16	0.90
Rate of Fe(II) oxidation (mM day^−1^)	0.26	0.15	0.19	0.23
Rate of Fe(III) reduction (mM day^−1^)	0.16	0.18	—^*a*^	—^*a*^
Rate of NO_3_^−^ reduction (mM day^−1^)	0.31	0.14	0.083	0.064
Stoichiometry of NO_3_^−^_reduced_/Fe(II)_oxidized_	1.23	0.89	0.44	0.28

a No Fe(III) reduction was measured.

b Amount of nitrate that was reduced while Fe(II) was oxidized.

The second phase of the experiment was initiated by the readdition of 2 mM Fe(II) after 103 days of incubation, when readily available organic carbon was depleted and the nitrate concentration remained constant at ca. 3.6 mM. The extent and the rate of Fe(II) oxidation in phase 2 were comparable to those for the first cycle in phase 1 (Table 2 and Fig. 2 and 3). No changes in Fe(II) concentrations were measured in NaN_3_-inhibited controls.

**FIG 4:**
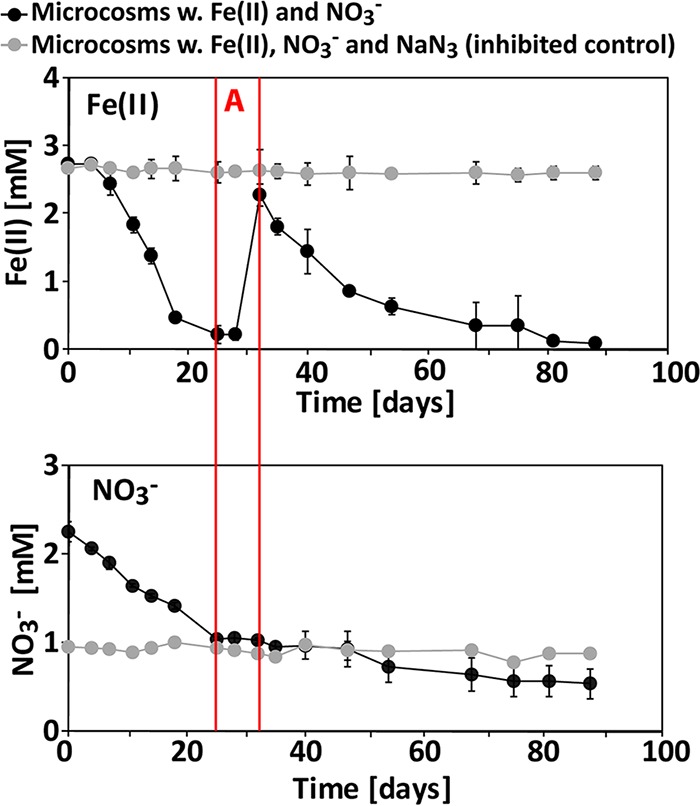
Development of Fe(II) and NO_3_^−^ concentrations in a microcosm experiment with sediment from Kalø Vig. Shown are data for total Fe(II) (extraction with 40 mM sulfamic acid) and NO_3_^−^ in the dissolved phase. The time interval “A,” marked by red lines in the graphs, shows that nitrate reduction had ceased because oxidizable Fe(II) was depleted, but nitrate reduction started again at day 32 when fresh Fe(II) was added. Error bars show standard deviations of data from triplicates (in some cases, they are smaller than the symbol).

In the microcosm incubations of sediment from Kalø Vig, the Fe(II) concentration in the 40 mM sulfamic acid extract decreased continuously over the first 25 days and remained constant at ca. 0.2 mM (Fig. 4) until day 32. At that point, 2 mM FeCl_2_ was readded to the microcosms, and Fe(II) oxidation continued until day 88. The amounts of Fe(II) oxidized and the calculated Fe(II) oxidation rates are shown in Table 3. No changes in Fe(II) concentrations were observed in the NaN_3_-inhibited controls.

**TABLE 3:** Decreases of Fe(II) and NO_3_^−^ concentrations and rates and stoichiometries of Fe(II) oxidation and NO_3_^−^ reduction at different time intervals in the microcosm experiment with sediment from Kalø Vig

Time of incubation (days)	Decrease of Fe(II) concn (mM) [rate of Fe(II) oxidation (mM day^−1^)]	Decrease of NO_3_^−^ concn (mM) (rate of NO_3_^−^ oxidation [mM day^−1^])	Stoichiometry of NO_3_^−^_reduced_/Fe(II)_oxidized_
0–25	2.5 (0.10)	1.25 (0.05)	0.49
32–88	2.17 (0.039)	0.47 (0.008)	0.22

### Nitrate and nitrite concentrations in microcosms over time.

The temporal development of the NO_3_^−^ concentrations in microcosms from Norsminde Fjord is shown in Fig. 2 and 3. In the first phase of the experiment, NO_3_^−^ was completely consumed in the first and second redox cycles after day 12 and day 50 (Fig. 2). In the third redox cycle, however, only 1.16 mM NO_3_^−^ was reduced until day 71, and from that time point on, the NO_3_^−^ concentration remained constant at 3.6 ± 0.05 mM until day 103, when Fe(II) was replenished. The rates of NO_3_^−^ reduction decreased throughout the duration of the experiment (Table 2). Nitrite accumulated only during the first Fe redox cycle in phase 1, where it reached a maximum concentration of 0.62 ± 0.23 mM after 12 days. During the following cycles, maximum nitrite concentrations were <50 μM (see Fig. S2 in the supplemental material). In the NaN_3_-inhibited controls, NO_3_^−^ concentrations remained constant over time, and no accumulation of NO_2_^−^ was observed (Fig. 2; see also Fig. S2 in the supplemental material).

In the microcosms with sediment from Kalø Vig, NO_3_^−^ concentrations decreased except between days 25 and 32, when concentrations remained constant (Fig. 4). The rates of NO_3_^−^ reduction were higher at the beginning of the incubation than toward the end (Table 3). NO_2_^−^ was found to accumulate only between days 4 and 35, with a maximum concentration of 0.27 mM after 11 and 14 days (see Fig. S3 in the supplemental material). In the NaN_3_-inhibited controls, NO_3_^−^ concentrations remained constant over time, and no accumulation of NO_2_^−^ was observed (Fig. 4; see also Fig. S3 in the supplemental material).

### Stoichiometries of NO_3_^−^_reduced_ versus Fe(II)_oxidized_.

The ratio of reduced NO_3_^−^ to oxidized Fe(II) changed significantly during the microcosm incubation experiment. In microcosms with sediment from Norsminde Fjord, 1.23 mol of NO_3_^−^ was initially reduced for every mole of Fe(II) oxidized (Table 2). The ratio decreased throughout the experiment, and during the second phase of the experiment, only 0.28 mol of NO_3_^−^ was reduced per mol of Fe(II) oxidized. Similarly, in microcosms with sediment from Kalø Vig, the ratio of reduced NO_3_^−^ to oxidized Fe(II) changed from initially 0.49 to 0.22 over time (Table 3).

### ^14^C incorporation into biomass during nitrate-reducing Fe(II) oxidation.

**FIG 5:**
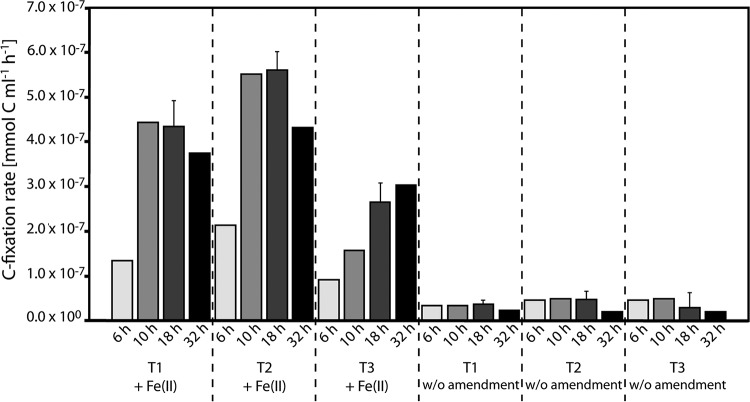
C fixation rates measured in mini-microcosms during three time intervals in phase 2 of the experiment. Rates from incubations where Fe(II) was added (NO_3_^−^ was still present after preincubation) are shown on the left. Rates from incubations where no Fe(II) was added and also no consumption of NO_3_^−^ was observed over time (NO_3_^−^ was still present after preincubation) are shown on the right. Error bars show standard deviations of data for three samples (available for the 18-h samples only).

To demonstrate the activity of autotrophic nitrate-reducing Fe(II)-oxidizing bacteria, we quantified the incorporation of ^14^C-labeled bicarbonate into biomass during the second phase of the experiment, i.e., when we saw Fe(II) oxidation coupled to nitrate reduction after organic carbon depletion. We found that background ^14^C incorporation in the control microcosms that contained NO_3_^−^ but no Fe(II) amendment was about 5 times higher (3.8 × 10^−8^ ± 1.1 × 10^−8^ mmol of C ml^−1^ h^−1^) than that in the NaN_3_-inhibited controls (7.2 × 10^−9^ ± 1.1 × 10^−8^ mmol of C ml^−1^ h^−1^) (Fig. 3). The addition of Fe(II) to the microcosms increased the rate of ^14^C incorporation to 3.8 × 10^−7^ ± 1.6 × 10^−7^ mmol of C ml^−1^ h^−1^ (Fig. 5), i.e., ∼10-fold higher than that of the Fe-free controls and 50-fold higher than that of the NaN_3_-inhibited controls (Fig. 3).

### Stoichiometries of Fe(II)_oxidized_ per C_fixed_.

To determine how many moles of Fe(II) were oxidized by autotrophic nitrate-reducing Fe(II) oxidizers to fix 1 mol of CO_2_, we plotted the amount of Fe(II) oxidation against the amount of CO_2_ fixation during the second phase of the experiment and applied linear regression. The linear regression showed that 26.5 mol of Fe(II) was oxidized per mol of fixed CO_2_ (*R*^2^ = 0.85) (see Fig. S4 in the supplemental material).

## DISCUSSION

### Transition from heterotrophic denitrification and chemodenitrification to autotrophic Fe(II) oxidation coupled to nitrate reduction.

This study shows evidence of the presence of autotrophic nitrate-reducing Fe(II) oxidizers in sediment from Norsminde Fjord and Kalø Vig. We quantified the contributions of heterotrophic nitrate reduction versus autotrophic nitrate-reducing Fe(II) oxidation based on stoichiometries of NO_3_^−^_reduced_ versus Fe(II)_oxidized_. In microcosm experiments with both sediments, we found a decreasing activity of heterotrophic nitrate reduction over time due to the gradual depletion of readily available organic carbon. This was concluded based on the continuous decrease of the ratio of NO_3_^−^_reduced_ to Fe(II)_oxidized_ throughout the experiment. In microcosms from Norsminde Fjord and Kalø Vig, the ratios decreased from 1.23 and 0.49 at the beginning of the experiment to 0.28 and 0.22 at the end, respectively (Tables 2 and 3).

As can be seen from equation 1, a molar stoichiometry of 0.2 for reduced nitrate per Fe(II)_oxidized_ would be indicative of nitrate-reducing Fe(II) oxidation with N_2_ as the end product. However, some of the electrons from Fe(II) oxidation must be used for the reduction of CO_2_ (for biomass production) rather than the reduction of nitrate (for energy generation). In our microcosm experiments with sediment from Norsminde Fjord, 26.5 mol of Fe(II) was oxidized to fix 1 mol of carbon (Table 3). Based on this value, and the theoretical value of 4 mol of electrons that is needed to fix 1 mol of carbon, the stoichiometry of reduced nitrate per Fe(II)_oxidized_ for autotrophic nitrate-reducing Fe(II) oxidation should be 0.17.

We found initially high ratios of NO_3_^−^_reduced_ to Fe(II)_oxidized_ in the Norsminde Fjord microcosms. This implies that early nitrate reduction in the sediment microcosms was due primarily to heterotrophic nitrate reduction or mixotrophic nitrate-reducing Fe(II) oxidation. In the first phase of the experiment, even if all measured Fe(II) oxidation was microbially catalyzed, only 16, 23, and 45% of the measured nitrate reductions could be attributed to microbial nitrate-reducing Fe(II) oxidation in the first, second, and third redox cycles, respectively. However, the occurrence of chemodenitrification (58, 59) or Fe(III) reduction [which would replenish some Fe(II)] could lead to an over- or underestimation of the contribution of nitrate-reducing Fe(II) oxidizers, especially in the beginning of the microcosm incubations. Nitrite was formed only at the beginning of the incubation (accumulation of maximums of 0.62 mM after 12 days and 0.27 mM after 14 days in microcosms from Norsminde Fjord and Kalø Vig, respectively). After day 20 and day 25 for Norsminde Fjord and Kalø Vig microcosms, respectively, nitrite accumulation of >0.04 mM was no longer detected. As this shift in nitrite production is correlated with the expected shift of the relative contribution from mostly heterotrophic processes to an increasing importance of autotrophic processes, we assume that nitrite production in the presence of Fe(II) is linked to heterotrophic nitrate reduction, as outlined previously by Klueglein et al. (26).

### Bioavailability of Fe(II) and Fe extraction with sulfamic acid.

One of the main differences between the redox processes in the second phase and those in the first phase of the experiment was the concentration of nonoxidized Fe(II) in the 40 mM sulfamic acid extract. In the first phase, 0.9, 0.7, and 0.6 mM Fe(II) remained nonoxidized in redox cycles 1, 2, and 3, respectively. At these time points, when the total Fe(II) concentrations were not decreasing further, dissolved Fe(II) was no longer present (data not shown). In the second cycle, sufficient NO_3_^−^ (ca. 2 mM) was present (and also, NO_3_^−^ reduction continued), while no changes in Fe(II) concentrations (remaining at ca. 0.7 mM) in the 40 mM sulfamic acid extract were measured. This indicated that not all of the 40 mM sulfamic acid-extractable Fe(II) was available for microbial oxidation. In the second phase of the experiment, however, only 0.2 mM Fe(II) remained nonoxidized. The lower value of nonoxidized Fe(II) in phase 2 than that in phase 1 could be due to a higher crystallinity of Fe minerals in the microcosms in phase 2, leading to a lower extraction efficiency of 40 mM sulfamic acid. Alternatively, it could be due to increased Fe(II) bioavailability and Fe(II) oxidation caused by a change in Fe(II) mineralogy or a change/adaptation of the Fe(II)-oxidizing bacterial community during the transition from phase 1 to phase 2 caused by repeated Fe cycling.

### Nitrate consumption and potential formation of the N intermediates N_2_O and NO.

In the second phase of the Norsminde Fjord microcosm incubation, the NO_3_^−^_reduced_/Fe(II)_oxidized_ stoichiometry was 0.28. The calculated stoichiometry for autotrophic nitrate-reducing Fe(II) oxidation is 0.17 (see above). Taking this stoichiometry and the measured decrease in the Fe(II) concentration of 3.2 mM, we assign 0.55 mM the consumed NO_3_^−^ to Fe(II) oxidation. Since we measured a decrease in the NO_3_^−^ concentration of 0.9 mM, this yields 0.35 mM NO_3_^−^ disappearing that cannot be attributed to autotrophic nitrate-reducing Fe(II) oxidation. However, we analyzed only nitrate and nitrite concentrations in solution and no other N products or N intermediates of nitrate reduction. Several other processes could lead to a higher decrease in nitrate concentrations than expected; e.g., (i) nitrate reduction by heterotrophic or mixotrophic bacteria (despite the depletion of carbon during the first phase), (ii) storage of nitrate inside microbial cells, or (iii) incomplete reduction of nitrate to intermediate products such as NO or N_2_O could lead to an increased value for NO_3_^−^_reduced_ versus Fe(II)_oxidized_. In the control incubation, where no Fe(II) was added in the beginning of the second phase, no measurable decrease in the NO_3_^−^ concentration was observed. Therefore, it is unlikely that heterotrophic nitrate reduction occurred to any substantial degree in the microcosms to which Fe(II) was added. Also, even assuming the highest reported microbial storage capacities for nitrate (60, 61), with the low numbers of cells of nitrate-reducing Fe(II) oxidizers present, a substantial contribution of nitrate removal by intracellular storage is also unlikely. Therefore, the most likely explanation is that not all of the nitrate was reduced to N_2_, but rather, it was reduced only to N intermediates, such as N_2_O or NO, which is common for nitrate reducers (62, 63).

### Autotrophic nitrate-reducing Fe(II) oxidation depending on carbon availability.

The sediment from Norsminde Fjord contained around 3 wt% TOC. To detect the activity of autotrophic nitrate-reducing Fe(II) oxidizers, it was necessary to preincubate the sediment (phase 1) for >3 months to deplete the bioavailable organic carbon. The presence of bioavailable organic carbon and the resulting activity of heterotrophic nitrate-reducing bacteria, as well as possible abiotic side reactions, would have complicated ^14^C incorporation measurements in phase 1 for several reasons. First, it is likely that as long as there is still readily available organic carbon present, bacteria with the potential for autotrophic nitrate-reducing Fe(II) oxidation would instead grow heterotrophically or mixotrophically. Second, due to the activity of heterotrophic nitrate reducers in the first phase of the experiment, a significant fraction of Fe(II) oxidation in the beginning of the experiment could have happened via chemodenitrification by reaction with reactive nitrogen species produced by heterotrophic bacteria during nitrate reduction (58, 59). These abiotic reactions would have made it impossible to calculate the ratio of oxidized Fe(II) to fixed carbon. Third, carbon fixation by heterotrophic bacteria in anaplerotic reactions typically accounts for 5 to 10% of the total biomass produced by heterotrophic bacteria (64–66). This substantial amount of C fixation could have masked the expected low carbon fixation rates by autotrophic nitrate-reducing Fe(II) oxidizers at the early stages of the experiment.

However, after the long preincubation, we were able to detect and quantify autotrophic ^14^C fixation by nitrate-reducing Fe(II) oxidizers, although it is unclear whether these bacteria perform this process *in situ* in this organic-rich sediment. Our study rather suggests that a habitat with relatively high nitrate and Fe(II) concentrations combined with a low organic carbon content would be most suitable for studying the activity of autotrophic nitrate-reducing Fe(II) oxidizers. Under such conditions, they would not have to compete with heterotrophic nitrate reducers for nitrate, and because of organic carbon limitation, they would have a competitive advantage when living autotrophically. However, the organic carbon content should also not be too low. Otherwise, heterotrophic Fe(III) reduction would be limiting, and the nitrate-reducing Fe(II) oxidizers would have a smaller supply of Fe(II). Consequently, we expect that autotrophic nitrate-reducing Fe(II)-oxidizing bacteria play only a minor role in Fe(II) oxidation in the TOC-rich sediments of Norsminde Fjord. Indeed, the microcosm experiments with the low-TOC sediment from Kalø Vig (TOC concentration of 0.45 wt%) already showed evidence of autotrophic nitrate-reducing Fe(II)-oxidizing activity after a much shorter time than in experiments with the higher-TOC sediments from Norsminde Fjord. We therefore suggest that the Kalø Vig field site is suitable for further investigations of autotrophic nitrate-reducing Fe(II) oxidation. However, based on the low cell numbers for nitrate-reducing Fe(II) oxidizers that we determined by MPN analyses, it remains to be determined how relevant microbial nitrate-reducing Fe(II) oxidation is in the environment. A recent study by Jewell et al. (67) highlighted the importance of chemolithoautotrophic nitrate-reducing microorganisms in an oligotrophic groundwater environment. Based on metatranscriptomic analyses, those authors showed that after injection of nitrate, the abundances of Fe(II)-oxidizing Gallionellaceae and S-oxidizing Sulfurimonas spp. were increasing. Based on the limited knowledge about these microorganisms, it is difficult to predict or evaluate how important sulfide-oxidizing nitrate reducers are at the field sites that were investigated in the present study compared to Fe(II)-oxidizing nitrate reducers. At least in our microcosm incubations where sulfate reduction was inhibited, no sulfide was produced, and therefore, S-oxidizing nitrate reducers should not have been active.

### Fe(II) oxidation required for CO_2_ fixation and for energy generation.

We calculated that ca. 26 mol of Fe(II) was oxidized by nitrate-reducing Fe(II) oxidizers to fix 1 mol of carbon. To calculate how much energy is needed by nitrate-reducing Fe(II) oxidizers to fix 1 mol of carbon, we use a value of 1 mol of carbon fixation from CO_2_ that requires 4 mol of electrons (in the form of reducing equivalents, such as, e.g., NADH) and energy in the form of ATP. Consequently, if nitrate-reducing Fe(II) oxidizers oxidize 26.5 mol Fe(II) in order to fix 1 mol of carbon, and they need only 4 mol electrons/reducing equivalents for the fixation of 1 mol of carbon, they can use the other 22.5 mol of Fe(II) for energy generation.

From the estimated energy gain of −96.23 kJ/mol Fe(II)_oxidized_ (equation 1), we calculate that nitrate-reducing Fe(II) oxidizers need −2,165 kJ per mol CO_2_ fixed. This value is within the range reported previously for other chemolithoautotrophs such as aerobic sulfide oxidizers (68). Furthermore, we calculate that ∼15% of the electrons from Fe(II) are used for carbon fixation. This is also in good agreement with what was reported previously for other chemolithoautotrophs (usually 10 to 20% [69–72]). The molar ratios of the oxidized substrate to fixed carbon for other chemolithoautotrophs are, e.g., 2:1 to 5:1 for aerobic sulfur oxidizers (depending on the sulfur species and the electron acceptor [66, 72–74]), 10:1 for ammonium oxidizers (75, 76), and 25:1 to 80:1 for nitrite oxidizers (77). Calculating how many electrons these microbes need to transfer to fix 1 mol of CO_2_, this translates to a range of 16 to 40 for sulfur oxidizers, ca. 80 for ammonium oxidizers, and 50 to 160 for nitrite oxidizers. Thus, the amount of electrons transferred by nitrate-reducing Fe(II) oxidizers to fix 1 mol of C (ca. 26) is in the lower range and is most similar to what is known for aerobic sulfide oxidizers. The energy gain of sulfur oxidation per mole of electrons transferred is −99.25 kJ/mol (oxidation of sulfide to sulfate with oxygen as the electron acceptor [78]), which is similar to the energy yield of nitrate-reducing Fe(II) oxidizers (equation 1).

The values for oxidized Fe(II) per fixed C that we found for nitrate-reducing Fe(II) oxidizers are significantly lower than what was estimated previously for microaerophilic Fe(II) oxidizers, for which values of 43 to 70 mol oxidized Fe(II) per mol of fixed C were suggested (79, 80). However, these values probably contain large uncertainties due to the difficulties that are related to the accurate quantification of microbial microaerophilic Fe(II) oxidation rates, which compete with abiotic Fe(II) oxidation rates.

### Biomass production during autotrophic nitrate-reducing Fe(II) oxidation.

The rates of C fixation from all measurements in mini-microcosms that contained nitrate and Fe(II) were on average 2.9 × 10^−7^ ± 1.6 × 10^−7^ mmol C ml^−1^ h^−1^. Based on these C fixation rates, within the 14 days of the second phase of the experiment, there was ∼9.7 × 10^−4^ mmol C fixed per ml sediment slurry. This approximately equals the amount of C that is present in 5 × 10^6^ cells that could have been produced per ml of slurry (assuming 0.2 × 10^−12^ g per cell [dry weight] and a carbon content of 50% [dry weight]). However, this is only a rough estimation, and nitrate-reducing Fe(II)-oxidizing bacteria can produce substantial amounts of extracellular polymeric substances (EPSs) (26). Therefore, an unknown amount of the fixed carbon may not be going into the production of new cells but rather may be going to EPS production.

### Conclusions and outlook.

Our microcosm data on the stoichiometry of nitrate reduced per Fe(II) oxidized and ^14^CO_2_ incorporated provide evidence for the presence and activity of autotrophic nitrate-reducing Fe(II) oxidation in a marine sediment. Our study suggests that autotrophic nitrate-reducing Fe(II) oxidation is most likely to occur in sediments with low or intermediate organic carbon concentrations.

Our study provides the basis for several further investigations. In particular, it will now be possible to identify autotrophic nitrate-reducing bacteria in the Norsminde Fjord and Kalø Vig sediments, e.g., by DNA-SIP (stable isotope probing). Additionally, if it is known which bacteria are oxidizing Fe(II) autotrophically, this will assist in isolation attempts, as it can be evaluated whether these bacteria or closely related bacteria have special demands, e.g., regarding vitamins or growth conditions. Finally, in environments that are likely to support the activity of autotrophic nitrate-reducing Fe(II) oxidizers *in situ*, such as the Kalø Vig field site, the general environmental relevance of this process can be determined.

## Supplementary Material

Supplemental material
