# Supplementary material for: Evidence for the Existence of Autotrophic Nitrate-Reducing Fe(II)-Oxidizing Bacteria in Marine Coastal Sediment
Source: Appl Environ Microbiol. 2016 Sep 30;82(20):6120–31. doi: 10.1128/AEM.01570-16 (PMC5068159; doi:10.1128/AEM.01570-16)
Supplement: Supplemental material [file supp_82_20_6120__index.html]

Supplemental material 

# Evidence for the Existence of Autotrophic Nitrate-Reducing Fe(II)-Oxidizing Bacteria in Marine Coastal Sediment

## Supplemental material

- Supplemental file 1 -

  Schematic illustration of the wet fumigation method applied in this study to quantify 14C assimilation by autotrophic nitrate-­reducing Fe(II) oxidizers (Fig. S1); development of nitrite concentrations over time in microcosms with Norsminde sediment (Fig. S2); development of nitrite concentrations in a microcosm experiment with a sediment with low TOC content (Kalø Vig, Denmark) (Fig. S3); graph used for calculation of the stoichiometry of Fe(II) oxidized per C fixed (Fig. S4); effect of the addition of NaN3 to enrichment cultures of nitrate-reducing Fe(II) oxidizers and Fe(III) reducers from Norsminde Fjord and Kalø Vig sediment (Table S1).

  PDF, 553K
