# Supplementary material for: Evidence for the Existence of Autotrophic Nitrate-Reducing Fe(II)-Oxidizing Bacteria in Marine Coastal Sediment
Source: Appl Environ Microbiol. 2016 Sep 30;82(20):6120–31. doi: 10.1128/AEM.01570-16 (PMC5068159; doi:10.1128/AEM.01570-16)
Supplement: Supplemental material [file AEM.01570-16_zam999117455so1.pdf]

Evidence for the existence of autotrophic nitrate-reducing Fe(II)-oxidizing bacteria in marine coastal sediment

*Katja Laufer[1], Hans Røy[2], Bo Barker Jørgensen[2], Andreas Kappler[1,2]**

[1]Geomicrobiology, Center for Applied Geosciences, University of Tübingen, Germany

[2]Center for Geomicrobiology, Department of Bioscience, Aarhus University, Denmark

*To whom correspondence should be sent:

Andreas Kappler, Geomicrobiology, Center for Applied Geosciences

University of Tuebingen, Sigwartstrasse 10, D-72076 Tuebingen, Germany

Phone: +49-7071-2974992, Fax: +49-7071-29-295059

Email: andreas.kappler@uni-tuebingen.de

Running title: Evidence for autotrophic nitrate-reducing Fe(II) oxidation

Supplementary Information

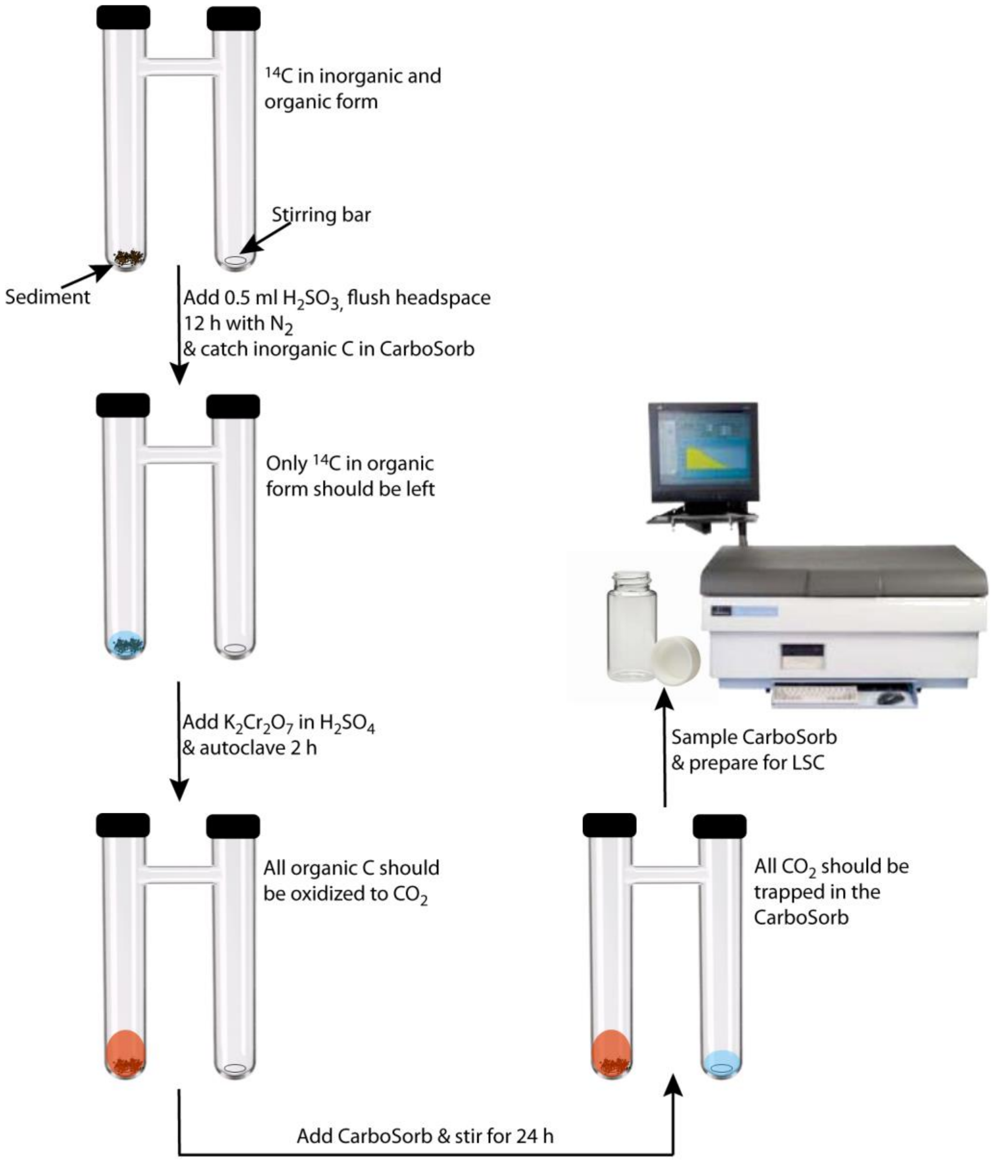

Figure S1: Schematic illustration of the wet fumigation method applied in this study to quantify ^{14}C-assimilation by autotrophic nitrate-reducing Fe(II)-oxidizers.

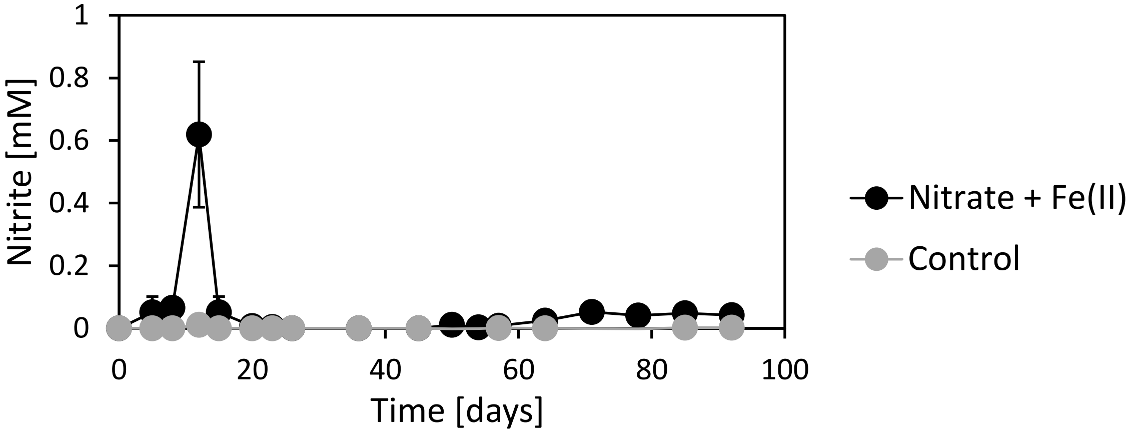

Figure S2: Development of nitrite concentrations over time in microcosms with Norsminde sediment. Black symbols show biotic samples, to which nitrate (4 mM) and Fe(II) (2 mM) were added and in which nitrate reduction and Fe(II) oxidation were observed. Grey symbols show abiotic controls, to which, in addition to Fe(II) (2 mM) and nitrate (4 mM), also NaN_3 (final concentration of 160 mM) was added.

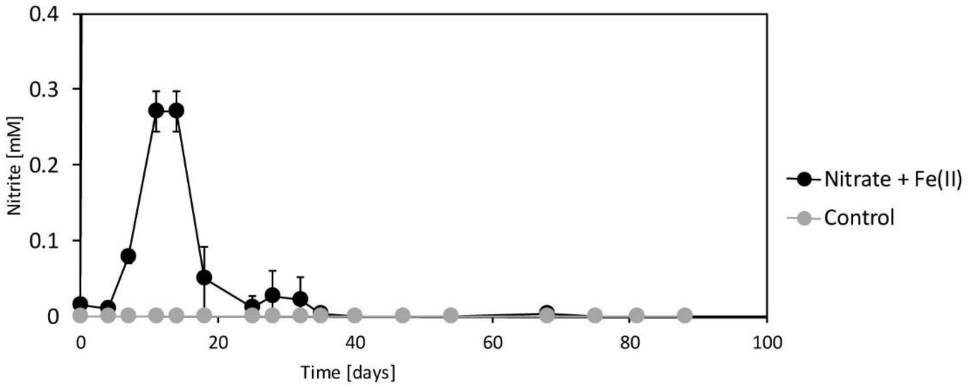

Figure S3: Development of nitrite concentrations in a microcosm experiment with a sediment with low-TOC content (Kalø Vig, Denmark).

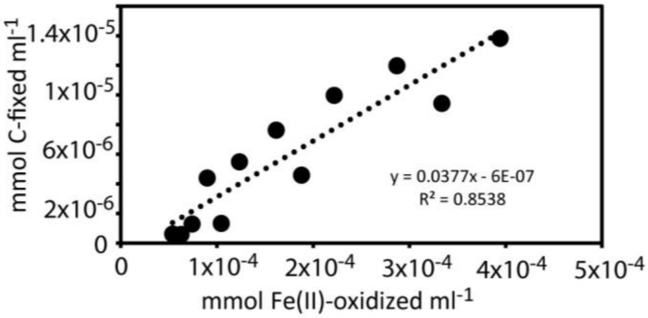

Figure S4: Graph used for calculation of the stoichiometry of Fe(II)-oxidized per C-fixed. Plotted were the amounts of C-fixed per ml against the amount of Fe(II)-oxidized per ml. A linear regression (dashed-line) was applied to calculate the stoichiometry. The equation and the R^2 of the regression are shown in the graph.

Table S1: Effect of the addition of NaN_3 to enrichment cultures of nitrate-reducing Fe(II)-oxidizers and Fe(III)-reducers from Norsminde Fjord and Kalø Vig sediment. The Fe(II) concentrations were determined by the spectrophotometric ferrozine assay. Nitrate concentrations were determined by flow injection analysis. Cellular growth was determined by fluorescence microscopy.

Enrichment culture	NaN_3 concentration	Fe(II) concentration at day 0	NO_3^- concentration at day 0	Fe(II) concentration at day 14	NO_3^- concentration at day 14	Cellular growth after 14 days
NO_3^--reducing Fe(II)-oxidizers Norsminde Fjord	0 mM	9.86 mM	4.14 mM	3.24 mM	1.47 mM	Yes
	1 mM	9.97 mM	2.05 mM	9.89 mM	2.12 mM	No
	10 mM	9.54 mM	1.91 mM	9.64 mM	1.85 mM	No
	160 mM	9.81 mM	2.03 mM	9.98 mM	2.98 mM	No
NO_3^--reducing Fe(II)-oxidizers Kalø Vig	0 mM	9.79 mM	3.87 mM	2.93 mM	0.74 mM	Yes
	1 mM	9.86 mM	2.16 mM	9.91 mM	2.13 mM	No
	10 mM	9.98 mM	2.07 mM	10.01 mM	1.98 mM	No
	160 mM	9.83 mM	2.17 mM	9.89 mM	2.07 mM	No

Enrichment culture	NaN_3 concentration	Fe(III) concentration at day 0	Fe(III) concentration at day 14	Cellular growth after 14 days
Fe(III)-reducers Norsminde Fjord	0 mM	5.57 mM	0.02 mM	Yes
	1 mM	5.23 mM	5.19 mM	No
	10 mM	4.89 mM	4.92 mM	No
	160 mM	5.36 mM	5.31 mM	No
Fe(III)-reducers Kalø Vig	0 mM	5.29 mM	0.08 mM	Yes
	1 mM	6.07 mM	5.99 mM	No
	10 mM	4.96 mM	4.84 mM	No
	160 mM	4.78 mM	4.82 mM	No
